# Effectiveness of nail bed repair in children with or without replacing the fingernail: NINJA multicentre randomized clinical trial

**DOI:** 10.1093/bjs/znad031

**Published:** 2023-03-22

**Authors:** Abhilash Jain, Aina V. H. Greig, Amy Jones, Cushla Cooper, Loretta Davies, Akiko Greshon, Heidi Fletcher, Adam Sierakowski, Melina Dritsaki, Thi Thu An Nguyen, May Ee Png, Jamie R. Stokes, Helen Dakin, Jonathan A. Cook, David J. Beard, Matthew D. Gardiner, NINJA Collaborative, A. Mertic, H. Gerrish, K. Cranmer, N. Fox, P. Dutta, G. Vissers, P. Costa, R. Irri, G. McArthur, M. Horwitz, A. Sleiwah, H. Jephson, M. Deeley, R. Nicholas, Z. Vinnicombe, A. Nicola, C. Bing Chuo, C. Milner, J. Heaney, J. Totty, M. Fleet, M. Faheem Khadim, P. Williams, S. Bibawy, A. Round, R. Pinder, A. Plonczak, G. Lawton, D. Kennedy, A. Bennett, A. Fadulelmola, J. James, E. Reay, K. Beadon, T. Cameron, Z. Oliver, K. Wensley, S. Dupré, J. Rodriguez, D. Furniss, M. Gale, A. Knight, J. Tulip, L. Turner, L. Wellings, M. Allen, R. Wade, V. Itte, G. Bourke, N. Kumar, S. O’Sullivan, J. WM Jones, K. Young, K. Taylor, O. Dawood, S. Booth, L. Giwa, R. Pearl, A. Coutts, R. Hawkins, A. Mostafa, T. Nisbett, P. Riddlestone, A. Selby, C. Uzoho, D. Chasiouras, LC. Bainbridge, T. Buick, W. Lam, B. Baker, K. Walsh, K. Keating, R. Dalan, M. Shah, D. Mead, S. Diment, M. Nicolau, B. Smeeton, D. Thomson, N. Senior, J. Moledina, J. Colville, K. Manso, M. Song, O. Manley, P. Drury, R. Kerstein, W. Cobb, J. Wormald, R. Shirley, A. Tan, A. Arnaout, C. Cruz, N. Brice, N. Segaren, N. Joji, R. Chawla, S. Hassanin, R. Adami, H. Ridha, A. Cook, L. Symington, R. Long, S. Dustagheer, H. Jarvis, M. Larsen, M. Williams, R. Trickett, D. Miles, A. Pai, C. Honeywell, C. Brady, S. Madhavan, V. Manou, G. Phillips, R. Baker

**Affiliations:** Broomfield Hospital, Chelmsford, UK; Broomfield Hospital, Chelmsford, UK; Broomfield Hospital, Chelmsford, UK; Broomfield Hospital, Chelmsford, UK; Broomfield Hospital, Chelmsford, UK; Chelsea and Westminster Hospital, London, UK; Chelsea and Westminster Hospital, London, UK; Chelsea and Westminster Hospital, London, UK; Chelsea and Westminster Hospital, London, UK; Chelsea and Westminster Hospital, London, UK; Guys and St Thomas’ Hospitals, London, UK; Guys and St Thomas’ Hospitals, London, UK; Guys and St Thomas’ Hospitals, London, UK; Guys and St Thomas’ Hospitals, London, UK; Guys and St Thomas’ Hospitals, London, UK; Guys and St Thomas’ Hospitals, London, UK; Hull Royal Infirmary, Hull, UK; Hull Royal Infirmary, Hull, UK; Hull Royal Infirmary, Hull, UK; Hull Royal Infirmary, Hull, UK; Hull Royal Infirmary, Hull, UK; Hull Royal Infirmary, Hull, UK; Hull Royal Infirmary, Hull, UK; Hull Royal Infirmary, Hull, UK; Hull Royal Infirmary, Hull, UK; Hull Royal Infirmary, Hull, UK; St Mary’s Hospital, London, UK; St Mary’s Hospital, London, UK; St Mary’s Hospital, London, UK; James Cook Hospital, Middleborough, UK; James Cook Hospital, Middleborough, UK; James Cook Hospital, Middleborough, UK; James Cook Hospital, Middleborough, UK; John Racliffe Hospital, Oxford, UK; John Racliffe Hospital, Oxford, UK; John Racliffe Hospital, Oxford, UK; John Racliffe Hospital, Oxford, UK; John Racliffe Hospital, Oxford, UK; John Racliffe Hospital, Oxford, UK; John Racliffe Hospital, Oxford, UK; Kings Mill Hospital, Sutton in Ashfield, UK; Leeds General Infirmary, Leeds, UK; Leeds General Infirmary, Leeds, UK; Leeds General Infirmary, Leeds, UK; Leeds General Infirmary, Leeds, UK; Leeds General Infirmary, Leeds, UK; Leeds General Infirmary, Leeds, UK; Leeds General Infirmary, Leeds, UK; Leeds General Infirmary, Leeds, UK; Peterborough City Hospital, Peterborough, UK; Peterborough City Hospital, Peterborough, UK; Peterborough City Hospital, Peterborough, UK; Queen Victoria Hospital, East Grinstead, UK; Queen Victoria Hospital, East Grinstead, UK; Queen Victoria Hospital, East Grinstead, UK; Queen Victoria Hospital, East Grinstead, UK; Queen Victoria Hospital, East Grinstead, UK; Queen Victoria Hospital, East Grinstead, UK; Royal Cornwall Hospital, Truro, UK; Royal Cornwall Hospital, Truro, UK; Royal Cornwall Hospital, Truro, UK; Royal Cornwall Hospital, Truro, UK; Royal Cornwall Hospital, Truro, UK; Royal Derby Hospital, Derby, UK; Royal Derby Hospital, Derby, UK; Royal Derby Hospital, Derby, UK; Royal Derby Hospital, Derby, UK; Royal Hopsital for Sick Children, Edinburgh, UK; Royal Hopsital for Sick Children, Edinburgh, UK; Royal Manchester Children’s Hospital, Manchester, UK; Royal Manchester Children’s Hospital, Manchester, UK; Royal Manchester Children’s Hospital, Manchester, UK; Royal Manchester Children’s Hospital, Manchester, UK; Royal Manchester Children’s Hospital, Manchester, UK; Salisbury District Hospital, Salisbury, UK; Salisbury District Hospital, Salisbury, UK; Salisbury District Hospital, Salisbury, UK; St George’s Hospital, London, UK; St George’s Hospital, London, UK; St George’s Hospital, London, UK; St George’s Hospital, London, UK; St George’s Hospital, London, UK; Stoke Mandeville Hospital, Aylesbury, UK; Stoke Mandeville Hospital, Aylesbury, UK; Stoke Mandeville Hospital, Aylesbury, UK; Stoke Mandeville Hospital, Aylesbury, UK; Stoke Mandeville Hospital, Aylesbury, UK; Stoke Mandeville Hospital, Aylesbury, UK; Stoke Mandeville Hospital, Aylesbury, UK; Stoke Mandeville Hospital, Aylesbury, UK; The Lister Hospital, Stevenage, UK; The Lister Hospital, Stevenage, UK; The Lister Hospital, Stevenage, UK; The Lister Hospital, Stevenage, UK; The Lister Hospital, Stevenage, UK; The Lister Hospital, Stevenage, UK; The Lister Hospital, Stevenage, UK; The Lister Hospital, Stevenage, UK; The Lister Hospital, Stevenage, UK; The Lister Hospital, Stevenage, UK; The Ulster Hospital, Belfast, UK; The Ulster Hospital, Belfast, UK; The Ulster Hospital, Belfast, UK; The Ulster Hospital, Belfast, UK; University Hospital of Wales, Cardiff, UK; University Hospital of Wales, Cardiff, UK; University Hospital of Wales, Cardiff, UK; University Hospital of Wales, Cardiff, UK; University of Essex, Colchester, UK; Wexham Park Hospital, Slough, UK; Wexham Park Hospital, Slough, UK; Wexham Park Hospital, Slough, UK; Wexham Park Hospital, Slough, UK; Wexham Park Hospital, Slough, UK; Wexham Park Hospital, Slough, UK; Wexham Park Hospital, Slough, UK; 1 Nuffield Department of Orthopaedics, Rheumatology and Musculoskeletal Sciences, University of Oxford, Oxford, UK; 2 Department of Plastic Surgery, Imperial College Healthcare NHS Trust, London, UK; 3 Department of Plastic Surgery, Guy’s and St Thomas’ NHS Foundation Trust, London, UK; 4 St Andrew’s Centre for Plastic Surgery and Burns, Mid and South Essex NHS Foundation Trust, Chelmsford, UK; 5 Oxford Clinical Trials Research Unit, Nuffield Department of Orthopaedics, Rheumatology and Musculoskeletal Sciences, University of Oxford, Oxford, UK; 6 Health Economics Research Centre, University of Oxford, Oxford, UK; 7 Department of Plastic Surgery, Frimley Health NHS Foundation Trust, Slough, UK

## Abstract

**Background:**

Surgery for nail bed injuries in children is common. One of the key surgical decisions is whether to replace the nail plate following nail bed repair. The aim of this RCT was to assess the clinical effectiveness and cost-effectiveness of nail bed repair with fingernail replacement/substitution compared with repair without fingernail replacement.

**Methods:**

A two-arm 1 : 1 parallel-group open multicentre superiority RCT was performed across 20 secondary-care hospitals in the UK. The co-primary outcomes were surgical-site infection at around 7 days after surgery and cosmetic appearance summary score at a minimum of 4 months.

**Results:**

Some 451 children presenting with a suspected nail bed injury were recruited between July 2018 and July 2019; 224 were allocated to the nail-discarded arm, and 227 to the nail-replaced arm. There was no difference in the number of surgical-site infections at around 7 days between the two interventions or in cosmetic appearance. The mean total healthcare cost over the 4 months after surgery was €84 (95 per cent c.i. 34 to 140) lower for the nail-discarded arm than the nail-replaced arm (*P* < 0.001).

**Conclusion:**

After nail bed repair, discarding the fingernail was associated with similar rates of infection and cosmesis ratings as replacement of the finger nail, but was cost saving. Registration number: ISRCTN44551796 (http://www.controlled-trials.com).

## Introduction

Nail bed injuries are the most common hand injury in children^1^. They are typically caused when a fingertip is crushed in a closing door. This results in displacement of the hard fingernail (nail plate) and laceration of the underlying soft nail bed. In the UK, 96 per cent of surgeons report surgically removing the nail plate, suturing the nail bed laceration, and replacing the nail plate on the nail bed^2^. Over 10 000 nail bed repair operations are performed each year in the UK, based on an estimate from a multicentre service evaluation^2^, and many more in the USA^3^, based on the incidence of the main causes of nail bed injury in children.

The rationale for replacing the nail plate includes protection of the repair, reduction of infection, less pain at dressing changes, and splinting of the nail fold to prevent synechiae. The Cochrane review^4^ investigating nail bed injuries found no randomized trials on nail bed repair type, and concluded there was a lack of evidence to inform key treatment decisions in the management of children’s fingertip injuries.

A pilot RCT informed the design and conduct of this definitive trial comparing replacing or discarding the fingernail after nail bed repair, and demonstrated that a large RCT was feasible^5^. The aim of this RCT was to assess whether discarding the fingernail during nail bed repair was superior to retaining it.

## Methods

The UK South Central Research Ethics Committee approved this study on 20 February 2018 (18/SC/0024).

### Trial design and participants

The NINJA (Nail bed INJury Analysis) trial was a multicentre, pragmatic two-arm parallel-group superiority RCT. The trial protocol and statistical and health economic analysis plans have been published^6,7^. The trial was overseen by independent steering and data and safety monitoring committees.

Participants were recruited from 20 UK National Health Service (NHS) hand surgery units. Inclusion criteria were: all children aged less than 16 years; a nail bed injury occurring within 48 h of presentation believed to require surgical repair; ability of patients, parents or guardians to give consent to inclusion and complete follow-up; and injury to a single finger. Patients were excluded if they had: an infected injury; underlying nail disease or deformity in the injured finger or contralateral finger before injury; a distal phalanx fracture requiring fixation; amputation; loss of part or all of the nail bed requiring reconstruction; and multiple nail bed injuries. Nail bed injuries extending to the nail fold were accepted.

### Interventions

#### Intervention 1: replace fingernail or substitute

Following debridement and suturing of the nail bed, the fingernail was replaced and secured with a figure-of-eight suture using Vicryl Rapide™ (Bridgewater, NJ) suture. A low-adherent dressing was applied. If the fingernail could not be replaced (for example owing to damage or loss), a substitute was chosen by the operating surgeon (such as foil).

#### Intervention 2: discard fingernail

Following debridement and suturing of the nail bed, the fingernail was discarded, and a low-adherent dressing applied. The dressing was not used to splint open the nail fold.

### Outcomes

Baseline assessments were performed on the day of the operation before randomization, but after consent to participation had been provided. Follow-up assessments involved a clinical appointment between 7 and 10 days after operation, and a participant-reported questionnaire, sent via text, e-mail or post, at 7–10 days after operation, and at 4 months with a reporting window of up to 12 months.

### Co-primary outcome measures

#### Surgical-site infection at 7–10 days

The clinical research nurse or surgeon assessed the fingertip for evidence of surgical-site infection (SSI). Diagnosis of SSI was based on the presence of pain, swelling, tenderness, erythema, or purulent discharge. Definitive SSI had the addition of an organism isolated by culture or Gram stain^8^. Return to theatre for drainage of the nail bed wound and prescription of antibiotics were included as additional diagnostic markers of the participant having received treatment for a probable infection. Originally, the incidence of infection, defined in accordance with the Centers for Disease Control and Prevention criteria (CDC 1993) within 4 months, was selected. However, during the study set-up this was changed to the SSI at 7–10 days owing to concerns about attrition. This change was incorporated into version 1.0 of the protocol (5 December 2017).

### Cosmetic appearance of the nail

The cosmetic appearance of the fingernail was assessed using the Oxford Fingernail Appearance Score (OFNAS)^9^ at final follow-up, a minimum of 4 months up to 12 months after randomization. This score ranges from 0 (worst) to 5 (best nail appearance) with a single point awarded for each of the following features: subjective assessment of quality for nail shape, nail adherence, eponychium, nail surface, and nail plate split. The reference for assessment was the same finger on the uninjured contralateral hand. Any defects or reduction in quality of the nail compared with the contralateral side resulted in a score of 0 for that feature. Given the scale of changes and substantive differences in Zook score, on which the approach was originally based, the score was formalized as OFNAS in version 2.0 of the protocol (15 August 2018). It was originally intended to use OFNAS to score photographs of patients’ fingernails. Trainee surgeons who were blinded to the randomization group scored the returned photographs, and the median score across observers was used in the analysis. However, the proportion of returned photographs was considered too low (48.8 per cent). To improve the return rate, the team telephoned parents who had not responded and asked them to complete the score by answering the questions that make up the OFNAS. This change in follow-up strategy was formally incorporated into protocol version 3.0 (4 June 2019). It improved completeness of the outcome from 48.8 to 65.4 per cent.

### Secondary outcome measures

Secondary outcome measures collected were health-related quality of life using EuroQol Five Dimensions EQ-5D-Y™ (EuroQol Group, Rotterdam, the Netherlands), pain at first dressing change (measured using a 3-point Likert scale by children, participant or parent-assessed if the former was not able), SSI by 4 months, and participant (3-point Likert scale completed if able) and parent (0–100 scale) assessment of nail appearance at 4 months^6^. The EQ-5D-Y™ was completed by the child if aged 2 years or above (version appropriate for those aged either 2–6 or 7 years and over) at baseline, 7 days, and 4 months.

### Sample size

The sample size for NINJA was based on observed infection rates in previous studies and on the cosmetic outcome in the NINJA-P study. The sample size of 416 was based on a clinically important difference of 7 per cent in the proportion of patients with an SSI between the two treatment groups, as well as a 15 per cent difference between treatment groups in the proportion of those achieving an optimal result in terms of the cosmetic appearance of the nail. These differences were chosen to provide 90 per cent power at a two-sided 5 per cent level of significance, with no adjustment made for multiple comparisons.

### Randomization and masking

A computer-generated sequence was used to randomize participants using an allocation ratio of 1 : 1. Randomization was stratified according to recruitment site only, and treatment group numbers were balanced using sequences of random permuted blocks of sizes 2 and 4. Randomization was undertaken by a member of the research team once the participant had reached the operating theatre. Neither the surgeons nor participants could be masked. The cosmetic outcome assessment was performed by masked independent researchers.

### Statistical analysis

Statistical analyses followed the statistical and health economic analysis plan^7^. They were conducted on an as-randomized basis, irrespective of compliance. Analyses were on a complete-case basis unless stated otherwise. A two-sided significance level of 0.05 was used for all tests carried out with the corresponding 95 per cent confidence interval calculated accordingly. Data were summarized by group using appropriate summary statistic measures (for example mean(s.d.)).

The co-primary outcome SSI was analysed using logistic regression, adjusting for recruitment site as the only stratification factor using the cluster robust option in Stata^®^ (StataCorp, College Station, TX, USA) for the main analysis. The co-primary outcome OFNAS for each participant was calculated as the median of the scores given by five independent reviewers assessing photographs of the injured fingertips. The probability that discarding the nail was the optimal treatment over replacement was also calculated by Mann–Whitney U test analysis with 95 per cent confidence interval using the ranksum command in Stata^®^. This was calculated instead of the median as originally planned^7^, which was decided before calculating the respective quantities.

The cost-effectiveness analysis took a time horizon of up to 12 months and estimated the cost per infection avoided, using the primary outcome measure from the trial (infections at 7 days). The cost of operating time was included. Further information about the cost-effectiveness analysis is provided in the statistical and health economic analysis plan, and detailed methods and results of the economic evaluation will be reported separately. Confidence intervals were estimated using bootstrapping and included multiple imputation of missing data. Cost is presented in Euros based on an exchange rate of 1 GBP = 1.126 EUR on 8th February 2023.

### Patient and public involvement

Patients and the public were involved from the start of the programme of research during the development of the initial pilot study and this definitive RCT. A parent/patient survey helped to set the primary and secondary outcomes, for example the decision to have co-primary outcomes of infection and cosmetic appearance. A patient representative was a trial co-investigator, a member of the trial management group, and involved at every stage of study design and delivery. They provided advice on the burden of participation, provision of patient information, and subsequent dissemination.

## Results

**Fig 1:**
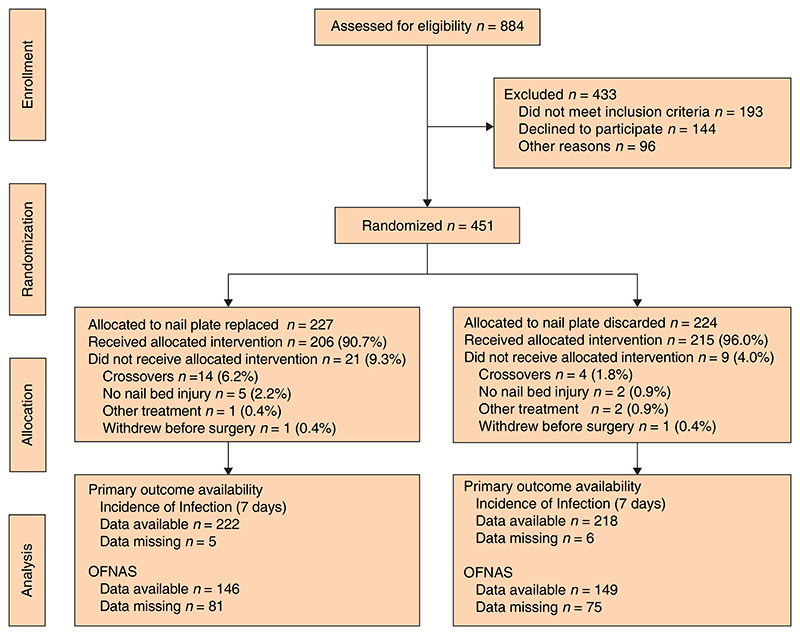
CONSORT diagram for the NINJA trial OFNAS,#Oxford Finger Nail Appearance Score

Some 451 patients were recruited between July 2018 and December 2019; 224 patients were allocated to the nail-discarded arm and 227 to the nail-replaced arm (Figure 1, Table 1 and Fig. S1). Compliance with allocation was high (96.0 and 90.7 per cent respectively) (Table 2). Baseline characteristics, including quality-of-life scores and age group proportions, were similar in the two groups. Boys and girls were evenly split in the nail-discarded group, but there were more boys in the nail-replaced group. Most patients had crush injuries and avulsions of the nail plate, and proportions of each injury type were similar in the two groups. One patient in each group withdrew from the study before surgery.

The type of anaesthetic used was similar in each group, as were perioperative antibiotics (Table S1). Most patients had povidone– iodine as antiseptic surgical preparation in both groups, with a slightly higher proportion in the nail-discarded group. Most patients received either 6/0 or 7/0 interrupted Vicryl Rapide™ sutures, with similar proportions of patients receiving either none or other types of suture. Most patients experienced some other injury in addition to the nail bed injury. In 25 of 213 patients (11.7 per cent) who had a nail replaced, a substitute (typically foil) was used instead of the patient’s nail.

Two serious adverse events were recorded. The first patient developed paronychia (infection around the nail plate) approximately 4 months after surgery, requiring readmission to hospital and removal of a nail spicule. This was an expected adverse event and determined to be related to the surgical intervention. The second patient suffered a reaction to general anaesthetic causing vomiting and intolerance to anything orally, leading to a 1-week stay in hospital for hydration. This was an unexpected adverse event and was deemed unlikely to be related to the surgical intervention.

For the co-primary outcome SSI, 440 patients had data available for the primary analysis (97.6 per cent of those randomized; 218 in discarded arm, 222 in replaced arm) (Table 3). Seven SSIs were recorded as part of the co-primary outcome (all in participants who complied with the intervention), five in the nail-replaced and two in the nail-discarded group. The adjusted analysis of the infection co-primary outcome produced an OR of 2.49 (95 per cent c.i. 0.58 to 10.61; *P* = 0.218), indicating that replacement of the fingernail was more closely associated with the infection, but this was not statistically significant.

Assessors and parents scored patients’ fingernails using the OFNAS for the cosmetic co-primary outcome (Table S2). Both assessor and parent scores were heavily skewed towards the positive end of the scale, with the modal score being 5 among patients who received an assessment (Table 4).

There was no significant difference in cosmetic appearance between groups in the co-primary outcome of cosmetic appearance, as measured by the OFNAS (*P* = 0.118, Mann–Whitney U test). The probability that a score randomly selected from the discard group was higher than a score randomly selected from the replace group was 0.55 (95 per cent c.i. 0.49 to 0.60). Owing to the later inclusion of parent cosmetic appearance assessments (to assist with trial conduct), it was decided to perform a post hoc subgroup analysis to determine whether the scores given by the assessors and parents differed between treatment groups (Table 4).

The adjusted secondary analysis did not identify any statistically significant difference in OFNAS values between the two treatment groups (OR 0.70, 95 per cent c.i. 0.43 to 1.12; *P* = 0.138).

The assessor scores did not indicate a difference between the nail-replaced and nail-discarded groups. However, the scores given by the parents suggested that there was a statistically significant difference in favour of the nail-discarded group. The treatment by subgroup interaction term was statistically significant (OR 0.24, 95 per cent c.i.: 0.06, 0.96. *P* = 0.044).

Preoperative antibiotic use did not affect the difference in cosmetic appearance between the two treatment groups as measured by the OFNAS (Table 4). The treatment by subgroup interaction term was not statistically significant (OR 1.11, 0.62 to 2.31; *P* = 0.805). Sensitivity analyses were carried out on each co-primary outcome, neither of which suggested sensitivity to missing data (Fig. S2 and Table S3).

There were no statistical differences between groups for the secondary outcomes, including pain at dressing change, late incidence of SSI (up to 12 months), parent/child satisfaction with nail appearance, and EQ-5D-Y™ index scores (Table S4). There were no statistically significant differences in any of the secondary outcomes.

## Economic evaluation

The base-case economic evaluation showed that the mean NHS cost in the first 4–12 months after nail bed repair surgery was £75.07 (95 per cent c.i. 30.05 to 124.11) higher in the replace group than the discard group (*P* < 0.001). The majority of the cost difference was attributable to the additional suture (assumed cost £4.27) and 3.4 (95 per cent c.i. 1.3, 5.1) min longer operating time required for nail replacement (Table S1).

After multiple imputation of missing data, the mean incidence of infections by 7–10 days was 0.0137 (95 per cent c.i. −0.0091 to 0.0352) per patient higher in the replace group than in the discard group (*P* = 0.338). Because the discard group had significantly lower costs and numerically fewer infections, the policy of discarding the fingernail dominated the policy of replacing the fingernail.

## Discussion

The NINJA trial showed no statistically significant difference in early (day 7) infections or final cosmetic outcome between patients who had the fingernail replaced and those who had the fingernail discarded after nail bed repair. The early infection rate (day 7) was 2.2 per cent in the nail-replaced group versus 0.9 per cent in the nail-discarded group. At 4 months’ follow-up the infection rate in the nail-replaced group increased to 3.5 per cent and remained at 0.9 per cent in the nail-discarded group. On the basis of infection, replacing the nail is not justified.

There was no difference between groups in cosmetic outcome or satisfaction with appearance, suggesting that replacing the nail, or not, does not influence the appearance of the new nail that grows out.

There was no evidence that replacing the nail after surgery can offer reduced pain at dressing change. A slightly larger number of children experienced pain in the nail-discarded group (47.8 per cent) compared with the nail-replaced group (40.5 per cent) but this did not reach statistical significance.

The health economic analysis showed that replacing the nail was associated with significantly longer operating time and cost. This resulted in a statistically significant cost saving of £75 per patient if the nail was discarded. Nail bed repair is the commonest paediatric hand trauma operation performed globally; this represents a significant cost saving to healthcare systems. As 96 per cent of nail bed repair procedures currently involve replacement of the nail^2^, the NHS could save £720 000 per year each year in the UK if nails were discarded in all 10 000 operations conducted.

The NINJA trial is the first large RCT investigating outcomes after paediatric fingertip injury, and provides strong evidence to direct practice. Development of the OFNAS has provided a more evidence-based tool for assessing nail cosmesis for future studies. The infection rate was lower than anticipated in both groups, and will have contributed to the lower level of statistical precision. The very low rate of missing data at 7–10 days for the infection rate is a great strength, but the later missing data at the final cosmesis time point is a limitation. However, the sensitivity analysis which looked at the potential impact of missing data (using the RCTmiss command) suggested the finding was robust. A further limitation was the necessary changeover of cosmetic assessment method to include parents reporting a large proportion of the final assessment.

Compared with the published literature, this study provides more robust data on the potential harms and likely outcome of patients undergoing nail bed repair. This will support the counselling of parents and older children. Previous non-randomized studies reported higher rates of infection perhaps owing to the limitations of non-randomized retrospective studies^10^.

Whether to replace or discard the nail plate has been a longstanding source of contention among surgeons. This study has addressed this issue and laid the foundation for asking a further question: Should the nail bed be repaired or not? Nail bed injuries encompass a wide spectrum of injury severities. For instance, a sizeable proportion might be amenable to being cleaned in the emergency department and dressed with adhesive strips.

## Figures and Tables

**Table 1:** Baseline characteristics of participants according to intervention group

	Nail replaced (*n* = 227)	Nail discarded (*n* = 224)	Total (*n* = 451)
**Age (years), mean(s.d.)**	5.7(3.8)	6.2(4.0)	5.9(3.9)
< 2	40 (17.6)	35 (15.6)	75 (16.6)
2–6	116 (51.1)	107 (47.8)	223 (49.4)
≥ 7	71 (31.3)	82 (36.6)	153 (33.9)
**Sex**			
F	94 (41.4)	115 (51.3)	209 (46.3)
M	133 (58.6)	109 (48.7)	242 (53.7)
EQ-5D™ index score, mean(s.d.)^*^			
2–6 years	0.7(0.3)	0.7(0.2)	0.7(0.3)
	(*n* = 79)	(n= 77)	(*n* = 156)
≥ 7 years	0.6(0.3)	0.6(0.3)	0.6(0.3)
	(*n* = 70)	(*n* = 79)	(*n* = 149)
**Injury type**			
Sharp lacerations	23 (10.1)	28 (12.5)	51 (11.3)
Stellate lacerations	4 (1.8)	2 (0.9)	6 (1.3)
Crush and avulsion of nail plate	185 (81.5)	173 (77.2)	358 (79.4)
Injuries involving sterile/germinal matrix	2 (0.9)	4 (1.8)	6 (1.3)
Pulp laceration and/or tuft fracture of distal phalanx	15 (6.6)	17 (7.6)	32 (7.1)

Values are n (%) unless otherwise indicated.

* No data on quality of life were collected for participants aged under 2 years. EQ-5D™, EuroQol Five Dimensions.

**Table 2:** Compliance with treatment allocated at randomization

	Nail replaced (*n* = 227)	Nail discarded (*n* = 224)
Received randomized treatment and nail bed repaired	206 (90.7)	215 (96.0)
Received randomized treatment but no nail bed injury to be repaired	5 (2.2)	2 (0.9)
Crossovers	14 (6.2)	4 (1.8)
Received alternative procedure	1 (0.4)	2 (0.9)
Withdrawn before surgery	1 (0.4)	1 (0.4)

Values are n (%) unless otherwise indicated.

**Table 3:** Analysis of surgical-site infection at around 7 days

	Nail replaced (*n* = 222)	Nail discarded (*n* = 218)	Unadjusted model		Adjusted model	
			OR^*^	*P*	Adjusted OR^*^	*P*
Surgical-site infection	5 (2.2)	2 (0.9)	2.49 (0.48, 12.97)	0.279	2.49 (0.58, 10.61)	0.218
Missing	5 (2.2)	6 (2.7)				

Values are n (%) unless otherwise indicated;

* values in parentheses are 95% confidence intervals.

**Table 4:** Main, secondary, and subgroup analyses of Oxford Finger Nail Appearance Score cosmetic outcome

	Nail replaced	Nail discarded	Effect size^*^	P^§^
**Main analysis**				
OFNAS, median (i.q.r.)	5 (4–5)	5 (4–5)	0.55 (0.49, 0.60)^†^	0.118^¶^
**Secondary analyses**				
Adjusted			0.70 (0.43, 1.12)^‡^	0.138
Unadjusted			0.70 (0.44, 1.10)	0.118
**Subgroup analyses**				
Assessor (parent *versus* child)			0.24, (0.06, 0.96)^‡^	0.044
Preoperative antibiotic use			1.11 (0.62, 2.31)^‡^	0.805

* Values in parentheses are 95% confidence intervals; effect sizes are shown as ORs, except

† probability that Oxford Finger Nail Appearance Score (OFNAS) in discard arm is greater than that in replace arm from Mann–Whitney U test.

‡ Adjusted model allowed for intrasite correlation using cluster-robust standard errors.

§ Ordinal regression, except

¶ Mann–Whitney U test.

## Data Availability

Requests to access the dataset from qualified researchers trained in human subject confidentiality protocols may be sent to SITU at situ@ndorms.ox.ac.uk.
