# Supplementary material for: Effectiveness of nail bed repair in children with or without replacing the fingernail: NINJA multicentre randomized clinical trial
Source: Br J Surg. 2023 Mar 22;110(4):432–8. doi: 10.1093/bjs/znad031 (PMC7614411; doi:10.1093/bjs/znad031)
Supplement: znad031_Supplementary_Data [file znad031_supplementary_data.docx]

**Effectiveness of nail bed repair in children with or without replacing the fingernail: the NINJA multicentre randomised controlled trial**

Abhilash Jain^1,2^*

Aina V H Greig^3^*

Amy Jones^1^

Cushla Cooper^1^

Loretta Davies^1^

Akiko Greshon^1^

Heidi Fletcher^1^

Adam Sierakowski^4^

Melina Dritsaki^1^ ORCID ID 0000-0002-1673-3036

Thi Thu An Nguyen^1^

May Ee Png^1^

Jamie R Stokes^5^ ORCID ID 0000-0002-5279-2332

Helen Dakin^6^ ORCID ID 0000-0003-3255-748X

Jonathan A. Cook^1^ ORCID ID 0000-0002-4156-6989

David J. Beard^1^ ORCID ID 0000-0001-7884-6389

Matthew D. Gardiner^1,7^ ORCID ID 0000-0002-8058-4186

NINJA Collaborative

*joint first authors

1. Nuffield Department of Orthopaedics, Rheumatology and Musculoskeletal Sciences, University of Oxford, Oxford, United Kingdom
2. Department of Plastic Surgery, Imperial College Healthcare NHS Trust, London, United Kingdom
3. Department of Plastic Surgery, Guy’s and St Thomas’ NHS Foundation Trust, London, United Kingdom
4. St Andrew’s Centre for Plastic Surgery and Burns, Mid and South Essex NHS Foundation Trust, Chelmsford, United Kingdom
5. Oxford Clinical Trials Research Unit, Nuffield Department of Orthopaedics, Rheumatology and Musculoskeletal Sciences, University of Oxford, Oxford, United Kingdom
6. Health Economics Research Centre, University of Oxford, Oxford, United Kingdom
7. Department of Plastic Surgery, Frimley Health NHS Foundation Trust, Slough, United Kingdom

**Corresponding author.** Matthew D. Gardiner, Nuffield Department of Orthopaedics, Rheumatology and Musculoskeletal Sciences, University of Oxford, Oxford, United Kingdom. **ORCID I**D 0000-0002-8058-4186

**Supplementary Materials - Index**

| **Supplementary Figures and Tables** |  |
| --- | --- |
| Figure S1 | *pag. 4* |
| Figure S2 | *pag. 5* |
| Table S1 | *pag. 6* |
| Table S2 | *pag. 7* |
| Table S3 | *pag. 8* |
| Table S4 | *pag. 9* |
|  |  |

**Supplementary Figures and Tables**

### Figure S1: CONSORT diagram showing participant flow through trial

|  |  | Screened for eligibility  **n = 884** | |  |  |
| --- | --- | --- | --- | --- | --- |
|  |  |  |  |  |  |
|  |  |  |  | Ineligible **n = 193** | |
|  |  |  |  | Declined **n = 144** | |
|  |  |  |  | Other **n = 96** | |
|  |  |  |  |  |  |
|  |  | Randomised  **n = 451** | |  |  |
|  |  |  |  |  |  |
|  |  |  |  |  |  |
| Nail plate replaced  **n = 227** | |  |  | Nail plate discarded  **n = 224** | |
|  |  |  |  |  |  |
| Received allocated treatment:  **n = 206 (90.7%)**  Crossovers: **n = 14 (6.2%)**  No nail bed injury: **n = 5 (2.2%)**  Other treatment: **n = 1 (0.4%)**  Withdrew before surgery: **n = 1 (0.4%)** | |  |  | Received allocated treatment:  **n = 215 (96.0%)**  Crossovers: **n = 4 (1.8%)**  No nail bed injury: **n = 2 (0.9%)**  Other treatment: **n = 2 (0.9%)**  Withdrew before surgery: **n = 1 (0.4%)** | |
|  |  |  |  |  |  |
|  |  |  |  |  |  |
|  |  |  |  |  |  |
| **Primary outcome availability**  *Incidence of Infection (7 days)*  Data available: **n = 222** Data missing*:* ***n = 5***  *OFNAS Score*  Data available: **n = 146** Data missing*:* ***n = 81*** | |  |  | **Primary outcome availability**  *Incidence of Infection (7 days)*  Data available: **n = 218** Data missing: ***n = 6***  *OFNAS Score*  Data available: **n = 149** Data missing: ***n = 75*** | |

###
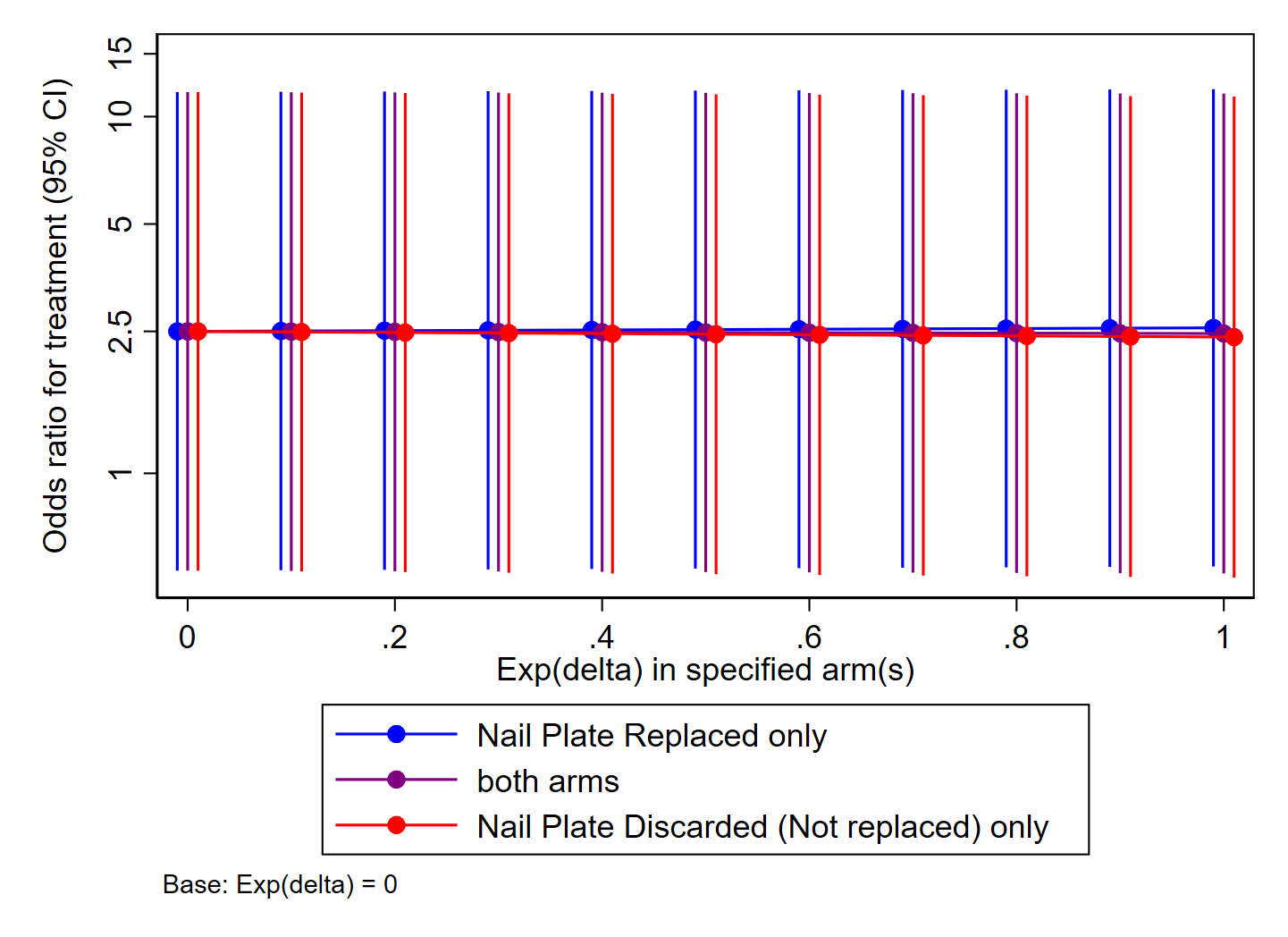
Figure S2: rctmiss sensitivity analysis for infection primary outcome

### Table S1: Details of operative procedures

|  | **Nail Replaced** | | **Nail Discarded** | | **Total** | |
| --- | --- | --- | --- | --- | --- | --- |
|  | **n** | **%** | **n** | **%** | **n** | **%** |
| **Total** | 225 | 49.9% | 223 | 49.4% | 448 | 99.3% |
| **Type of anaesthetic** | | | | | | |
| General | 47 | 20.9% | 44 | 19.7% | 91 | 20.3% |
| Local | 20 | 8.9% | 24 | 10.8% | 44 | 9.8% |
| Both | 157 | 69.8% | 155 | 69.5% | 312 | 69.6% |
| *Missing* | 1 | 0.4% | 0 | 0.0% | 1 | 0.2% |
| **Perioperative antibiotics** |  |  |  |  |  |  |
| Given | 99 | 44.0% | 94 | 42.2% | 193 | 43.1% |
| Not given | 126 | 56.0% | 127 | 57.0% | 253 | 56.5% |
| *Missing* | 0 | 0.0% | 2 | 0.9% | 2 | 0.4% |
| **Tourniquet type** | | | | | | |
| Finger | 183 | 81.3% | 185 | 83.0% | 368 | 82.1% |
| Upper arm | 7 | 3.1% | 5 | 2.2% | 12 | 2.7% |
| None used | 27 | 12.0% | 27 | 12.1% | 54 | 12.1% |
| *Missing* | 8 | 3.6% | 6 | 2.7% | 14 | 3.1% |
| **Antiseptic surgical prep used** | | | | | | |
| Povidone-iodine | 91 | 40.4% | 102 | 45.7% | 193 | 43.1% |
| Alcoholic chlorhexidine | 49 | 21.8% | 41 | 18.4% | 90 | 20.1% |
| Aqueous chlorhexidine | 72 | 32.0% | 67 | 30.0% | 139 | 31.0% |
| Other | 10 | 4.4% | 10 | 4.5% | 20 | 4.5% |
| *Missing* | 3 | 1.3% | 3 | 1.3% | 6 | 1.3% |
| **Surgical wash used** | | | | |  |  |
| Saline | 204 | 90.7% | 201 | 90.1% | 405 | 90.4% |
| Chlorhexidine | 5 | 2.2% | 4 | 1.8% | 9 | 2.0% |
| Betadine | 2 | 0.9% | 3 | 1.3% | 5 | 1.1% |
| Peroxide | 9 | 4.0% | 10 | 4.5% | 19 | 4.2% |
| Other | 3 | 1.3% | 1 | 0.4% | 4 | 0.9% |
| *Missing* | 2 | 0.9% | 4 | 1.8% | 6 | 1.3% |
| **Primary dressing** | | | | |  |  |
| Mepitel | 161 | 71.6% | 172 | 77.1% | 333 | 74.3% |
| Jelonet | 13 | 5.8% | 10 | 4.5% | 23 | 5.1% |
| Adaptic | 44 | 19.6% | 36 | 16.1% | 80 | 17.9% |
| Other | 6 | 2.7% | 5 | 2.2% | 11 | 2.5% |
| *Missing* | 6 | 2.7% | 5 | 2.2% | 11 | 2.5% |
| **Secondary dressing** | | | | |  |  |
| Boxing glove | 90 | 40.0% | 87 | 39.0% | 177 | 39.5% |
| Splint (finger based) | 21 | 9.3% | 17 | 7.6% | 38 | 8.5% |
| Other | 89 | 39.6% | 90 | 40.4% | 179 | 40.0% |
| *Missing* | 25 | 11.1% | 29 | 13.0% | 54 | 12.1% |
| **Sutures used** |  |  |  |  |  |  |
| 6/0 interrupted vicryl rapide | 154 | 68.4% | 162 | 72.6% | 316 | 70.5% |
| 7/0 interrupted vicryl rapide | 22 | 9.8% | 19 | 8.5% | 41 | 9.2% |
| Other | 33 | 14.7% | 24 | 10.8% | 57 | 12.7% |
| None used | 13 | 5.8% | 17 | 7.6% | 30 | 6.7% |
| *Missing* | 3 | 1.3% | 1 | 0.4% | 4 | 0.9% |
| **Nail bed injury type** |  |  |  |  |  |  |
| Laceration | 133 | 59.1% | 144 | 64.6% | 277 | 61.8% |
| Stellate laceration | 18 | 8.0% | 17 | 7.6% | 35 | 7.8% |
| Severe crush | 55 | 24.4% | 46 | 20.6% | 101 | 22.5% |
| Avulsion | 36 | 16.0% | 25 | 11.2% | 61 | 13.6% |
| *Missing^1^* | 16 | 7.1% | 13 | 5.8% | 29 | 6.5% |
| **Other injuries** |  |  |  |  |  |  |
| Adjacent skin injury | 83 | 36.9% | 79 | 35.4% | 162 | 36.2% |
| Tuft fracture | 27 | 12.0% | 24 | 10.8% | 51 | 11.4% |
| Adjacent skin injury + tuft fracture | 81 | 36.0% | 89 | 39.9% | 170 | 37.9% |
| *Missing* | 3 | 1.3% | 3 | 1.3% | 6 | 1.3% |
| **No nail bed/other injury** | 6 | 2.7% | 4 | 1.8% | 10 | 2.2% |
|  | **n** | **mean** | **SD** | **n** | **mean** | **SD** |
| **Operation time (minutes)** | 128 | 23.0 | 11.1 | 123 | 18.4 | 9.7 |

###

### Table S2: Oxford Finger Nail Appearance Score (OFNAS) median scores by treatment arm.

| **Component** | **Nail Replaced** | | **Nail Discarded** | | **Total** | |
| --- | --- | --- | --- | --- | --- | --- |
|  | **n** | **%** | **n** | **%** | **n** | **%** |
| **Total Score (Assessors)**^1^ |  |  |  |  |  |  |
| **0** | 0 | 0.0 | 1 | 0.4 | 1 | 0.2 |
| **1** | 1 | 0.4 | 1 | 0.4 | 2 | 0.4 |
| **2** | 3 | 1.3 | 3 | 1.3 | 6 | 1.3 |
| **3** | 7 | 3.1 | 10 | 4.5 | 17 | 3.8 |
| **4** | 41 | 18.1 | 32 | 14.3 | 73 | 16.2 |
| **5** | 59 | 26.0 | 62 | 27.7 | 121 | 26.8 |
| **Total Score (Parents)** |  |  |  |  |  |  |
| **0** | 0 | 0.0 | 0 | 0.0 | 0 | 0.0 |
| **1** | 0 | 0.0 | 0 | 0.0 | 0 | 0.0 |
| **2** | 2 | 0.9 | 0 | 0.0 | 2 | 0.4 |
| **3** | 1 | 0.4 | 2 | 0.9 | 3 | 0.7 |
| **4** | 13 | 5.7 | 4 | 1.8 | 17 | 3.8 |
| **5** | 19 | 8.4 | 34 | 15.2 | 53 | 11.8 |
| **Missing data** | 81 | 35.7 | 75 | 33.5 | 156 | 34.6 |
| 1. Scores are medians of multiple independent assessments | | | | | | |

### Table S3: Worst/best case sensitivity analysis for OFNAS co-primary outcome

|  | **Worst case (all missing=0)** | | **Best case (all missing=5)** | |
| --- | --- | --- | --- | --- |
|  | **Prob Discard > Replace^1^ 95% CI** | **P-value^2^** | **Prob Discard > Replace^1^ 95% CI** | **P-value^2^** |
| **Co-primary outcome** |  |  |  |  |
| OFNAS | 0.53 (0.48, 0.58) | 0.190 | 0.53 (0.48, 0.57) | 0.245 |
| 1. Probability OFNAS score in discard arm is greater than OFNAS score in replace arm  2. Mann-Whitney test | | | | |

**Table S4: Secondary outcome measures**

|  | **Nail Replaced (n=227)** | | | **Nail Discarded (n=224)** | | **Model Output** | | | |
| --- | --- | --- | --- | --- | --- | --- | --- | --- | --- |
|  |  |  |  | |  | ***Adjusted^1^*** | | ***Unadjusted*** | |
|  | ***n*** | ***%*** | ***n*** | | ***%*** | ***OR (95% CI)*** | | ***OR (95% CI)*** | |
| **Pain at dressing change at 7 days** |  |  |  | |  |  |  |  |  |
| Pain | 92 | 40.5 | 107 | | 47.8 | 0.71 (0.48, 1.04) | | 0.71 (0.48, 1.04) | |
| No pain | 123 | 54.2 | 101 | | 45.1 |  |  |  |  |
| **Surgical site infection at 4 months** |  |  |  | |  |  |  |  |  |
| Infection | 8 | 3.5 | 2 | | 0.9 | 4.39(0.96, 19.94) | | 4.39(0.92, 21.01) | |
| No infection | 135 | 59.5 | 148 | | 66.1 |  |  |  |  |
| **Satisfaction with nail appearance (child) at 4 months** |  |  |  | |  |  |  |  |  |
| Happy | 103 | 45.4 | 106 | | 46.7 | 1.47 (0.74, 2.94) | | 1.47(0.69, 3.15) | |
| Not happy or sad | 11 | 4.8 | 11 | | 4.8 |  |  |  |  |
| Sad | 7 | 3.1 | 2 | | 0.9 |  |  |  |  |
|  | ***n*** | ***mean (sd)*** | ***n*** | | ***mean (sd)*** | ***MD (95% CI)*** |  | ***MD (95% CI)*** |  |
| **Satisfaction with nail appearance (parent) at 4 months** | 121 | 80.5(26.0) | 120 | | 83.3(25.2) | -2.85(-10.17, 4.48) | | -2.85(-9.34, 3.64) | |
| **EQ-5D-Y at 7 days** |  |  |  | |  |  |  |  |  |
| Combined ages | 82 | 0.77(0.21) | 82 | | 0.75(0.25) | 0.03(-0.04, 0.10) | | 0.02(-0.05, 0.09) | |
| 2-6 years | 39 | 0.81(0.21) | 38 | | 0.80(0.25) |  |  |  |  |
| 7+ years | 43 | 0.73(0.21) | 44 | | 0.70(0.23) |  |  |  |  |
| **EQ-5D-Y at 4 months** |  |  |  | |  |  |  |  |  |
| Combined ages | 83 | 0.94(0.10) | 80 | | 0.97(0.08) | -0.02(-0.05, 0.00) | | -0.03(-0.05, 0.00) | |
| 2-6 years | 36 | 0.96(0.08) | 40 | | 0.99(0.03) | -0.02(-0.04, -0.00) | | -0.03(-0.07, 0.01) | |
| 7+ years | 47 | 0.93(0.12) | 40 | | 0.95(0.10) | -0.02(-0.06, 0.03) | | -0.02(-0.05, 0.02) | |

1. Models are adjusted for recruiting site with cluster-robust standard errors (EQ-5D-Y results are further adjusted for baseline EQ-5D-Y scores)
